# Microbiota-Dependent Upregulation of Bitter Taste Receptor Subtypes in the Mouse Large Intestine in High-Fat Diet-Induced Obesity

**DOI:** 10.3390/nu15194145

**Published:** 2023-09-25

**Authors:** Filippo Caremoli, Jennifer Huynh, Venu Lagishetty, Daniela Markovic, Jonathan Braun, Tien S. Dong, Jonathan P. Jacobs, Catia Sternini

**Affiliations:** 1 Division of Digestive Diseases, David Geffen School of Medicine at UCLA, Los Angeles, CA 90095, USA; filippo.caremoli@gmail.com (F.C.); huynh.jennifer.n@gmail.com (J.H.); vlagishetty@gmail.com (V.L.); tsdong@mednet.ucla.edu (T.S.D.); jjacobs@mednet.ucla.edu (J.P.J.); 2 Department of Medicine, David Geffen School of Medicine at UCLA, Los Angeles, CA 90095, USA; dmarkovic@mednet.ucla.edu; 3 Department of Neurobiology, David Geffen School of Medicine at UCLA, Los Angeles, CA 90095, USA; 4 Inflammatory Bowel and Immunobiology Institute, Cedars Sinai Medical Center, Los Angeles, CA 90048, USA; jonathan.braun2@cshs.org; 5 Division of Gastroenterology, Hepatology and Parenteral Nutrition, VA Greater Los Angeles Healthcare System, Los Angeles, CA 90073, USA

**Keywords:** antibiotics, dysbiosis, gut, microbiome, enteroendocrine cells, peptides, hormones

## Abstract

Bitter taste receptors (Tas2rs in mice) detect bitterness, a warning signal for toxins and poisons, and are expressed in enteroendocrine cells. We tested the hypothesis that Tas2r138 and Tas2r116 mRNAs are modulated by microbiota alterations induced by a long-term high-fat diet (HFD) and antibiotics (ABX) (ampicillin and neomycin) administered in drinking water. Cecum and colon specimens and luminal contents were collected from C57BL/6 female and male mice for qRT-PCR and microbial luminal 16S sequencing. HFD with/without ABX significantly increased body weight and fat mass at 4, 6, and 8 weeks. Tas2r138 and Tas2r116 mRNAs were significantly increased in mice fed HFD for 8 weeks vs. normal diet, and this increase was prevented by ABX. There was a distinct microbiota separation in each experimental group and significant changes in the composition and diversity of microbiome in mice fed a HFD with/without ABX. Tas2r mRNA expression in HFD was associated with several genera, particularly with *Akkermansia*, a Gram-negative mucus-resident bacterium. These studies indicate that luminal bacterial composition is affected by sex, diet, and ABX and support a microbial dependent upregulation of Tas2rs in HFD-induced obesity, suggesting an adaptive host response to specific diet-induced dysbiosis.

## 1. Introduction

The gustatory system acts as a gatekeeper that evaluates the quality of food and discriminates nutrients from potentially hazardous substances [1,2]. This chemosensory process starts in the mouth via the activation of receptors in taste buds, including taste receptors detecting sweet, umami, and bitter tastes that belong to the family of G protein-coupled receptors [3,4]. While sweet and umami tastes typically give hedonistic feedbacks, inducing acceptance behavior and influencing positively food intake, bitter taste serves as a warning mechanism to sense toxic or dangerous chemicals, which are often bitter, thus inducing avoidance or rejection responses [5,6]. Bitter taste is detected by a family of receptors (Tas2rs in mouse and TAS2Rs in humans), which includes 36 genes in rodents and 25 in humans [7,8]. Most Tas2rs/TAS2Rs can be activated by many chemically and structurally divergent substances, whereas others recognize a few or single compounds [9,10]. Tas2r/TAS2R expression has been reported in several sites in addition to the tongue, including the gastrointestinal (GI) tract, the lungs and respiratory tract, the reproductive systems, and the brain, providing evidence for non-gustatory functions of taste receptors outside the mouth [11,12,13,14,15,16].

The GI tract represents the largest surface that separates our body from the external environment, being constantly exposed to nutrients, microorganisms, and toxins [17]. Molecular sensing by specialized GI epithelial cells, including enteroendocrine cells (EEC), plays a critical role in the body homeostasis by regulating caloric intake and metabolism, insulin secretion, GI motility, and secretion [18,19,20]. EEC cells express chemoreceptors that detect intraluminal factors and release signaling molecules, which affect local or distant targets and activate neuronal pathways that in turn initiate functional responses. Tas2rs/TAS2Rs are expressed in the intestinal GI mucosa in rodents and humans [7,21], where they are localized to different types of epithelial cells [22,23,24,25,26,27,28,29]. We have shown that Tas2r138, which is selectively activated by thiourea/isothiocyanates such as phenylthiocarbamide (PTC), is highly expressed in the mucosa of the mouse large intestine, where it is localized to EEC cells [29], and its human counterpart, TAS2R38, is localized to different populations of EEC cells of the sigmoid colon, which include cells containing cholecystokinin (CCK), glucagon-like peptide 1 (GLP-1), and peptide YY (PYY) [24], peptides known to regulate different GI functions, satiety, and feeding behavior [18,19]. These findings, together with previous observations that intraluminal bitter tastants, including PTC, activate vagal afferents innervating the intestine and regulating GI functions [30], induce peptide release, and affect ingestive behavior [31,32,33], provide strong support to the hypothesis that Tas2rs/TAS2Rs play a role in gut chemosensory processes. Furthermore, our findings that Tas2r138 mRNA is upregulated in high-fat diet-induced obesity in mice [29] and that TAS2R38 mRNA and protein are upregulated in the colon of obese/overweight subjects [24] suggest that intraluminal changes in obesity affect these receptors’ expression. Diet is an important factor in modulating gut microbiota and impairment of intestinal microbiota has emerged as a trigger event in obesity and metabolic disorders [34,35,36].

The endogenous ligands for Tas2rs/TAS2Rs are still largely unknown but there is evidence that a subset of Tas2rs localized in rodent small intestine cells are activated by parasites to induce an immune response to infection [26] and that TAS2R38 in the respiratory system is broadly tuned for bacterial products [37,38,39], some of which have been detected in the gut lumen [40]. The presence of Tas2R/TAS2R subtypes in gut epithelial cells that produce antimicrobial peptides or mucous proteins, such as Paneth cells and goblet cells, respectively, provide additional evidence for a direct line of defense against external threats reaching the gut lumen [25,27]. Here, we tested the hypothesis that Tas2r subtypes in the large intestine of male and female mice are regulated by changes in the bacterial luminal contents induced by a long-term high-fat, high-caloric diet, which is known to result in obesity and dysbiosis [41,42,43] and antibiotic treatment that has been shown to cause perturbation of the gut microbiota [44,45,46]. We focused on two Tas2r subtypes, Tas2r138 and Tas2r116 that are activated by a different set of ligands and are expressed in the GI tract [8,9,33]. We found that both high-fat diet and antibiotics reduced the diversity and changed the composition of gut microbiota and that Tas2r138 and Tas2r116 mRNA levels were significantly increased only in high-fat diet without antibiotics and were significantly correlated with several bacteria. These data support a microbial dependent upregulation of Tas2rs in the large intestine, which might represent an adaptive response to obesity-induced dysbiosis.

## 2. Materials and Methods

*Animals and diets:* Male and female C57BL/6NCrL BR mice (Charles River Laboratory International, Inc, Hollister, CA, USA) 8 weeks of age were housed in a room at 23 ± 2 °C with a 12 h light-12 h dark cycle. Animal care and procedures were consistent with the humane use of animals as recommended by the National Institutes of Health. Experimental procedures were approved by the UCLA Animal Research Committee (ARC protocol 2015-011).

*Feeding manipulations:* Male and female mice were fed a high-fat diet (HFD) 60% fat, 5 kcal/g (Research Diets D12492) or standard chow food (normal diet, ND) 6.2% fat, 3.1 kcal/g (Teklad laboratory Diet, ENVIGO, Indianapolis, IN, USA), *ad libitum* with or without a combination of ampicillin, 1 g/L and neomycin, 0.5 g/L (VWR, Radnor, PA, USA), antibiotics with broad-spectrum, in drinking water for 8 weeks (8 male and 8 female mice fed ND, 8 male and 8 female mice fed ND with ABX, 8 male and 8 female mice fed HFD, and 8 male and 8 female mice fed HFD with ABX for a total of 64 mice, 32 males and 32 females; there were no deaths and no animals needed to be excluded). These diets match in protein composition and are comparable to diets used in previous studies from our and other groups [29,47,48]. Food, water intake, and weight were assessed bi-weekly and body composition (lean mass and fat mass) was measured at week 0 and by-weekly after week 4 until the end of the treatment, using Echo-MRI Scan (EchoMRI LLC., Houston, TX, USA). At the end of the treatments, mice were euthanized by isoflurane overdose between 8:00 a.m. and 12:00 p.m. Specimens from the large intestine (cecum and distal colon) and their luminal contents were collected from animals in each experimental condition. Tissue specimens were placed in RNAlater™ Stabilization Solution (Thermo Fisher Scientific, Waltham, WA, USA) and stored at 4 °C or −20 °C for qRT-PCR.

*RNA extraction and quantitative Real-Time Reverse Transcription Polymerae Chain Reaction (qRT-PCR):* Total RNA was isolated from the cecum and colon using Qiagen miRNeasy Mini Kit (Qiagen, Valencia, CA, USA) followed by DNase I treatment with Rnase-Free Dnase Set (Qiagen) to avoid genomic DNA contamination. RNA quality was estimated by the absorbance at 260 nm, 280 nm, and 230 nm ratios (OD260 nm/OD280 nm > 2.0, OD260 nm/OD230 nm > 1.7). Complementary DNA was generated using SuperScript^®^ III First-Strand Synthesis System (Invitrogen, Thermo Fisher Scientific) on a DNA Thermal Cycler Engine (BIO-RAD). qRT-PCR was performed using Taqman Gene expression MasterMix (ThermoFisher Scientific) for Tas2r138 (ThermoFisher Scientific, Mm01700131_s1) and Tas2r116 (ThermoFisher Scientific, Mm01160271_s1). Standard thermal cycles for Taqman Gene assays were run on Mx3000P Real-time PCR Detection System (Stratagene) and data were analyzed with Mx Pro 3000 software. β-actin (BA) was used as a housekeeping gene. The Delta Delta cycle threshold (Ct) method was used to calculate the relative abundance of mRNA expression [29]. Data were normalized to BA, and the control group for each experimental condition was set as internal standard. Samples were run at least in duplicate and No-RT and distilled RNAse-free water controls were always included.

*Peptides measurements:* The Bio-Plex Pro^TM^ Mouse Diabetes Multiplex Assay (Bio-Rad Laboratories, Hercules, CA, USA) is a magnetic bead-based (xMAP technology, Atlanta, GA, USA) multiplex assay that enables the simultaneous measurement of several protein biomarkers in serum or plasma and cell lysates and was used for this study. The assay was performed utilizing a Bio-Rad Bio-Plex 200^TM^ reader, and a Bio-Rad Bio-Plex Pro^TM^ Wash Station. Plasma samples were thawed on the same day of analysis and stored at 4 °C until testing. Mean peptide levels were compared by diet, sex, and antibiotic treatment using 3-way analysis of variance (ANOVA). The variables for GIP and insulin were log transformed to approximate the normal distribution. Multiple testing adjustments were performed using the Holm method.

*Blood plasma glucose and lipid panel measurement:* Postprandial amounts of glucose, total cholesterol (CHOL), high density lipoproteins cholesterol (HDL), and triglycerides (TRIG) in plasma were determined using the photometric system Vet Axcel^®^ Clinical Chemistry Analyzer (Alfa Wassermann Diagnostic Technologies, NJ, USA). Frozen samples were pipetted into sample cups and placed on the instrument for assessment. Low density lipoproteins (LDL_CA) values were estimated by the formula LDL_CA = CHOL − [(TRI/5) + HDL].

*16S ribosomal RNA sequencing:* Large intestine luminal contents were collected before storing the specimen in RNAlater by flushing it twice with 500 µL of sterile ddH_2_O. Samples were centrifuged at max speed at 4 °C. Pellets and supernatant from the luminal content were then separated into different tubes and stored at −80 °C. Cecal pellets underwent bacterial DNA extraction using the MO BIO Powersoil kit (Carlsbad, CA, USA). PCR amplification of the V4 region of the 16S ribosomal RNA gene was then performed to prepare a sequencing library according to a previously published protocol [49]. The resulting pooled library was sequenced using an Illumina MiSeq with the 2 × 150 bp v2 kit. QIIME v1.9.1 was used with default parameters to process the raw sequence data using closed reference operational taxonomic unit (OTU) picking against the Greengenes database at 97% identity (May 2013 version) [50]. The final depth ranged from 60,887 to 241,008 sequences per sample. Alpha diversity metrics (i.e., bacterial diversity within a sample) including Faith’s phylogenetic diversity metric, Chao1, and Shannon index were calculated in QIIME v1.9.1 using OTU-level data rarefied to 60,887 sequences. Significance was determined using the Mann–Whitney U test. Beta diversity analysis (i.e., differences in composition across samples) was performed using unweighted UniFrac distances with visualization by principal coordinates analysis. The significance of differences in beta diversity across groups was determined using Adonis, a permutational analysis of variance, with 100,000 permutations [51]. The association of OTUs with taste receptor expression was evaluated using negative binomial models implemented in the DESeq2 R package [52]. The models included sex and antibiotic/diet group as covariates; OTUs present in less than three samples were excluded from the analysis. *p*-values were adjusted for multiple hypothesis testing using the qvalue package in R, with q < 0.05 indicating significance [53].

*Statistical analysis:* We expressed values as the mean ± SEM. For multiple comparisons, we used one-way ANOVA and two-way ANOVA with Bonferroni post-test (*p* < 0.05 for significance). We utilized Prism v.8.4.1 (GraphPad Software, San Diego, CA, USA) for these statistical analyses. Peptides statistical analysis was performed as explained in the corresponding section. Specific statistical analysis for microbiome is described above and in figure legends.

## 3. Results

### 3.1. High-Fat Diet (HFD) Significantly Increases Body Weight and Fat Mass in Male and Female Mice

**Figure 1:**
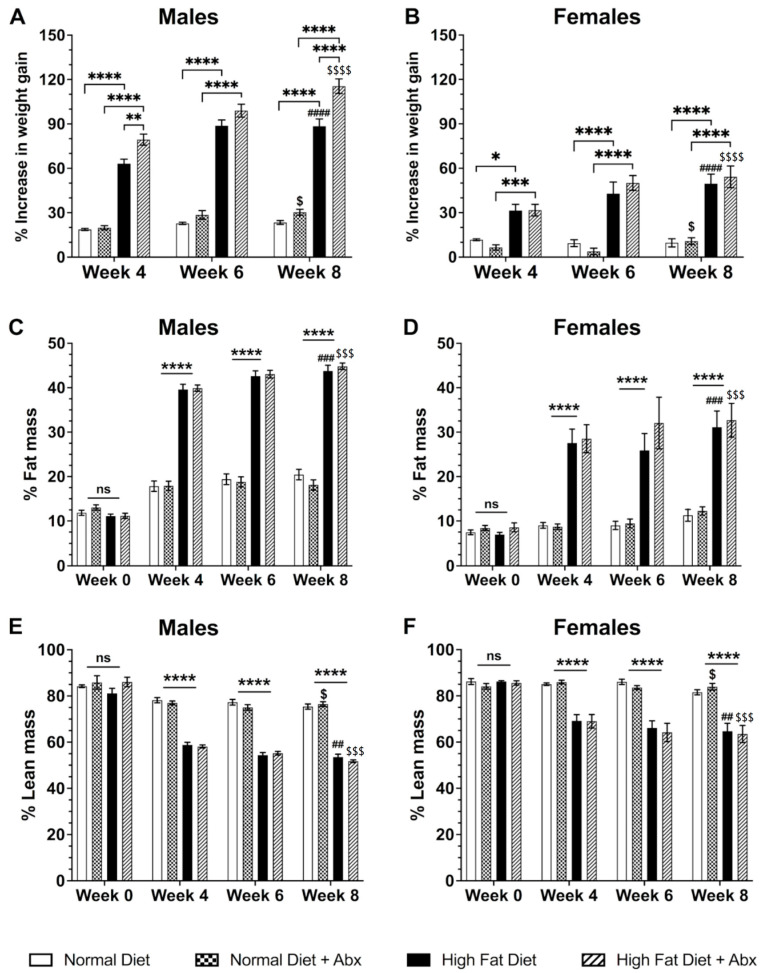
**Changes in body weight and body mass induced by high-fat diet (HFD) with or without antibiotics (Abx).** Body weight (expressed as % of increase from day 0) (**A**,**B**) and fat body mass (**C**,**D**) were significantly increased in male (**A**,**C**) and female (**B**,**D**) mice fed HFD with or without Abx treatment at 4, 6, and 8 weeks (* *p* < 0.05, ** *p* < 0.01, *** *p* < 0.001, **** *p* < 0.0001 vs. normal diet, ND). Body weight increase was higher in HFD with Abx compared to HFD without Abx only in males (**A**) (** *p* < 0.01, **** *p* < 0.0001). Body weight gain and fat mass increase were higher in males (**A**,**C**) vs. females (**B**,**D**) with HFD with Abx ($ *p* < 0.05, $$$ *p* < 0.001, $$$$ *p* < 0.0001) and without Abx (### *p* < 0.001, #### *p* < 0.0001). By contrast, lean body mass (**E**,**F**) was decreased in HFD with or without Abx in both males (**E**) and females (**F**) (**** *p* < 0.0001) compared to ND. However, lean mass in HFD with or without Abx was higher in females (**F**) compared to males (**E**), (## *p* < 0.01, $ *p* < 0.05, $$$ *p* < 0.001 with Abx). Finally, there was a significant difference in body weight and fat mass in ND with Abx ($ *p* < 0.05) between males and females.

Body weight in HFD with or without antibiotics was significantly increased at weeks 4, 6, and 8 compared to ND in males (*p* < 0.0001) and females (*p* < 0.05–0.0001) (Figure 1A,B). In the HFD groups, there was a significantly higher weight gain in male mice compared to female mice at 8 weeks in the absence or presence of ABX (*p* < 0.0001), and in males but not females treated with HFD + ABX compared to HFD alone at 4 and 8 weeks (Figure 1A,B) (*p* < 0.01–0.0001). HFD with and without ABX significantly increased the percentage of fat mass in both male and female mice at weeks 4, 6, and 8 (*p* < 0.0001), with male mice having a significantly higher percentage compared to female mice (*p* < 0.05–0.001) (Figure 1C,D). Conversely, lean mass decreased significantly in the groups of mice fed a HFD with and without ABX compared to groups fed a ND with or without ABX (*p* < 0.0001). The decrease in lean mass was significantly higher in males vs. females (*p* < 0.05–0.0001) (Figure 1E,F). There was no statistical difference in food and water intake at any time point between the HFD and ND fed mice.

### 3.2. Tas2R138 and Tas2R116 mRNA Expression in the Mouse Large Intestine

**Figure 2:**
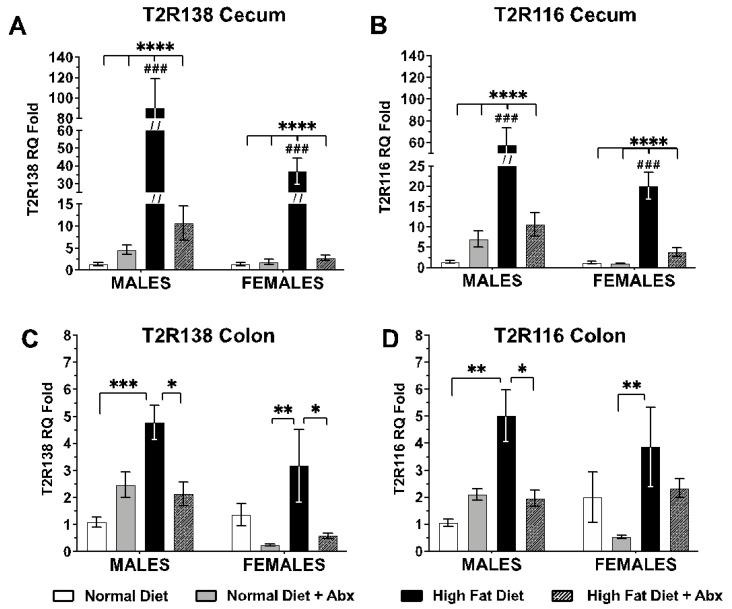
**Effect of high-fat diet (HFD) on Tas2r138 and Tas2r116 (labeled T2R138 and T2R116 in the figure) mRNA expression in the cecum and colon mucosa expressed as fold increase after 8 weeks of treatment**. qRT-PCR analysis showed Tas2r138 mRNA (**A**,**C**) and Tas2r116 mRNA (**B**,**D**) expression in the cecum ad colon. The levels of Tas2r138 and Tas2r116 mRNAs were markedly and significantly increased in the cecum of HFD without antibiotics (Abx) compared to ND with or without Abx and HFD with Abx (**A**,**B**) (**** *p* < 0.0001). Both Tas2r138 and Tas2r116 mRNA levels were significantly higher in HFD without Abx in males vs. females (### *p* < 0.001). In the male colon (**C**,**D**), both Tas2r138 and Tas2r116 mRNA were significantly higher in HFD than in ND alone (** *p* < 0.01; *** *p* < 0.001) and HFD + Abx (* *p* < 0.05). In the female colon, Tas2r138 and Tas2r116 mRNAs were significantly higher in HFD without Abx vs. ND with ABX (** *p* < 0.01). Tas2r138 was also significantly higher in HFD alone vs. HFD + Abx (* *p* < 0.05). *n* = 8 each treatment group, each sex.

A pilot study in male mice and cecum, the sex and region with the greatest increase of both Tas2rs following HFD at 8 weeks (Figure 2), showed that the levels of Tas2r138 and Tas2r116 mRNA in HFD-fed mice were not significantly different from those in ND-fed mice at 4 and 6 weeks with or without ABX (Supplementary Figure S1). Therefore, we focused our subsequent analysis of Tas2r mRNA expression, metabolic parameters, and microbiome at 8 weeks of treatment. qRT-PCR analysis showed a significant and marked increase in Tas2r138 and Tas2r116 mRNA levels in the cecum of HFD-fed mice (*p* < 0.0001 for males and females) vs. ND without ABX, which was significantly higher in males vs. females (*p* < 0.001) at 8 weeks (Figure 2A,B), with a lower but still significant increase of both Tas2r138 and Tas2r116 mRNAs in the colon of male mice (*p* < 0.01–0.001), whereas in female mice, the increase in both Tas2Rs reached statistical significance vs. ND or HFD with ABX but not vs. ND alone (*p* < 0.05–0.01) (Figure 2C,D); this was probably due to the higher variability observed in females vs. males in the colon of animals fed HFD alone. In addition, in the colon, there were no significant differences between males and females. Furthermore, the levels of Tas2r138 and Tas2r116 mRNAs in the cecum and colon of animals fed a HFD with ABX were comparable to ND with or without ABX. By contrast, there was no significant increase in Tas2r138 and Tas2r116 mRNAs in animals fed HFD with ABX (Figure 2A,B). The lack of significant differences in Tas2r138 and Tas2r116 mRNAs at 4 and 6 weeks of treatment suggests that the upregulation of Tas2r138 and Tas2r116 was a consequence of the intraluminal changes caused by the HFD, rather than a direct effect of the diet.

### 3.3. Metabolic Panel

**Figure 3:**
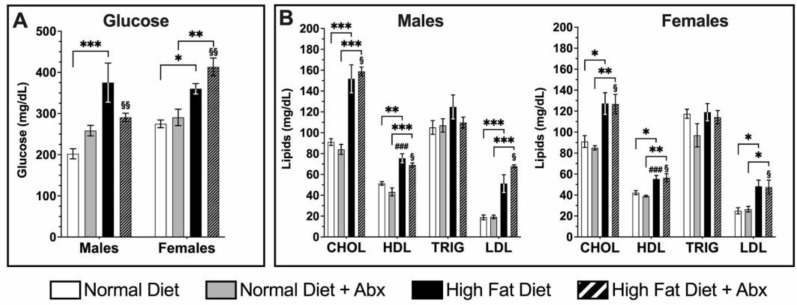
**Postprandial blood glucose and lipids levels in mice fed normal diet (ND) vs. high-fat diet (HFD) with or without antibiotics (Abx) at 8 weeks**. (**A**) Glucose levels in male and female mice fed HFD were significantly higher compared to ND mice (* *p* < 0.05 for females, *** *p* < 0.001 for males). Females fed HFD + Abx had higher glucose levels compared to males ($$ *p* < 0.01). Glucose levels were higher in females fed HFD + Abx compared to females fed ND + Abx (** *p* < 0.01). (**B**) Cholesterol (CHOL), high-density lipoproteins cholesterol (HDL), and low-density lipoproteins (LDL) were significantly higher in HFD groups compared to ND groups in both male and female mice (* *p* < 0.05, ** *p* < 0.01, *** *p* < 0.001 vs. ND control group). In HFD, HDL levels were higher in males vs. females (### *p* < 0.001). CHOL, HDL, and LDL levels in HFD + Abx were higher in males vs. females ($ *p* < 0.05). By contrast, triglycerides (TRIG) levels were comparable in HFD vs. ND with or without ABX. *n* = 4 each treatment group, each sex.

Postprandial glucose and lipids in blood plasma showed differences in both males and females fed a HFD compared to ND at 8 weeks, a time point that showed changes in Tas2R mRNA expression. Glucose levels were significantly higher in both males (*p* < 0.001) and females (*p* < 0.05) fed HFD compared to ND, whereas only female mice fed HFD with ABX showed a significant increase when compared to ND with ABX (*p* < 0.01) (Figure 3A). Glucose levels in females fed HFD with ABX were significantly higher than in the corresponding male group (*p* < 0.01) (Figure 3A). Total cholesterol (CHOL), high-density lipoprotein (HLD), and low-density lipoprotein (LDL) levels were significantly increased in HFD compared to ND with or without ABX (*p* < 0.05–0.001), whereas triglycerides (TRIG) remained comparable in each group. HDL levels were statistically higher in males vs. females in both HFD groups (*p* < 0.05–0.001), whereas CHOL and LDL levels were significantly higher in males vs. females (*p* < 0.05) only in the HFD + ABX group (Figure 3B).

**Figure 4:**
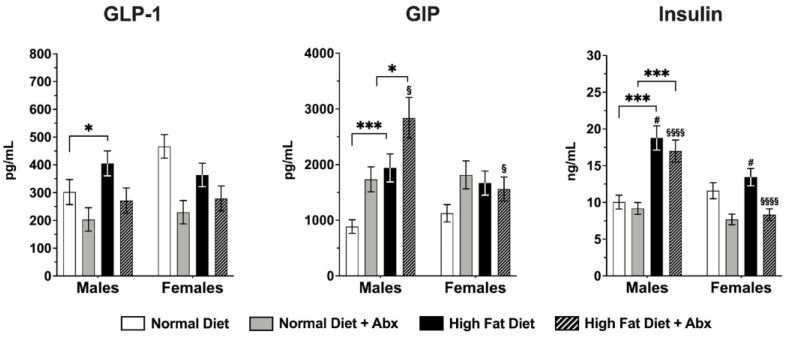
**Changes in hormones (GLP-1, GIP, and insulin) plasma levels in mice fed normal diet (ND) vs. high-fat diet (HFD) with or without antibiotics (Abx).** GLP-1 (* *p* < 0.05), GIP (*** *p* < 0.001), and insulin (*** *p* < 0.001) levels were significantly higher in males fed HFD compared to ND, but not in females. GIP levels in males fed HFD + Abx were significantly higher compared to males fed ND + Abx (* *p* < 0.05) and to females fed HFD + Abx ($ *p* < 0.05). Insulin levels in males fed HFD and HFD + Abx were also significantly higher than HFD fed females (# *p* < 0.05) and HFD + Abx fed females ($$$$ *p* < 0.0001), respectively. *n* = 8 per group.

Furthermore, there was a significant increase in the plasma levels of glucagon-like peptide-1 (GLP-1), glucose-dependent insulinotropic peptide (GIP), and insulin in male mice fed HFD vs. those fed ND (*p* < 0.05–0.001) but not in female mice at 8 weeks (Figure 4). GIP levels in males fed HFD + ABX were significantly higher than the same group in females (*p* < 0.05). Insulin levels in males fed HFD and HFD + ABX were also significantly higher than in HFD and HFD + ABX fed females (*p* < 0.05–0.0001) (Figure 4). Of note, GLP-1 levels in females fed ND were higher than in males fed ND, which might reflect different hormonal status of males and females [54].

### 3.4. Microbial Analyses

**Figure 5:**
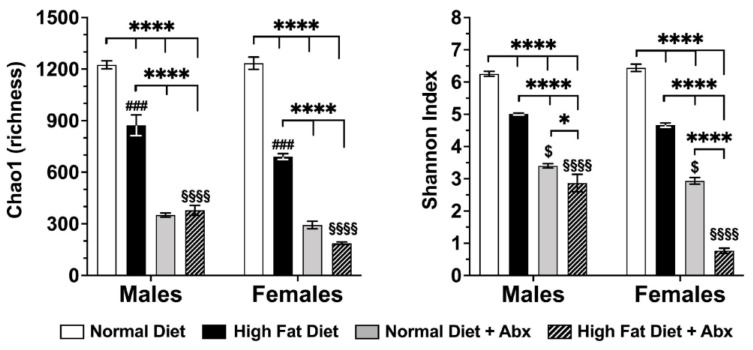
**Microbial α-diversity is decreased by high-fat diet (HFD) and by antibiotics (Abx).** Microbial diversity was assessed by Chao1 and Shannon index across different experimental groups in males and females. *n* = 8 each group, each sex. There was a significant decrease in α-diversity metrics in HFD vs. ND, which was even more pronounced by Abx with or without the HFD (**** *p* < 0.0001) in both Chao1 and Shannon indexes in males and females). There was significant difference in Shannon index (* *p* < 0.05 in males and **** *p* < 0.0001 in females) but not in Chao1 index between ND with Abx and HFD with Abx. The Chao1 index was significantly different in males and females fed a HFD (### *p* < 0.001). Finally, there was significance difference in Chao1 and Shannon indexes between males vs. females fed HFD with Abx (§§§§ *p* < 0.0001) and in Shannon but not Chao1 index between males vs. females fed ND with Abx ($ *p* < 0.05).

We focused this analysis on the luminal microbiota in the cecum, which had the highest levels of Tas2r138 and Tas2r116 mRNA expression in the mucosa following HFD. HFD caused a significant decrease of α-diversity metrics, including the Chao 1 index (a metric for species richness) and the Shannon index (a metric for species evenness and richness), in male and female mice (*p* < 0.0001 vs. ND) (Figure 5). ABX treatment caused a marked decrease in α-diversity metrics in both HFD and ND in males and females (*p* < 0.0001 vs. ND or HFD without ABX). The decrease in α-diversity metrics following HFD with ABX was more pronounced in females than in males for the Chao 1 index and Shannon index (*p* < 0.0001), whereas HFD alone caused a significantly more pronounced decrease in the Chao 1 index in females vs. males (*p* < 0.001) but not in the Shannon index (Figure 5).

**Figure 6:**
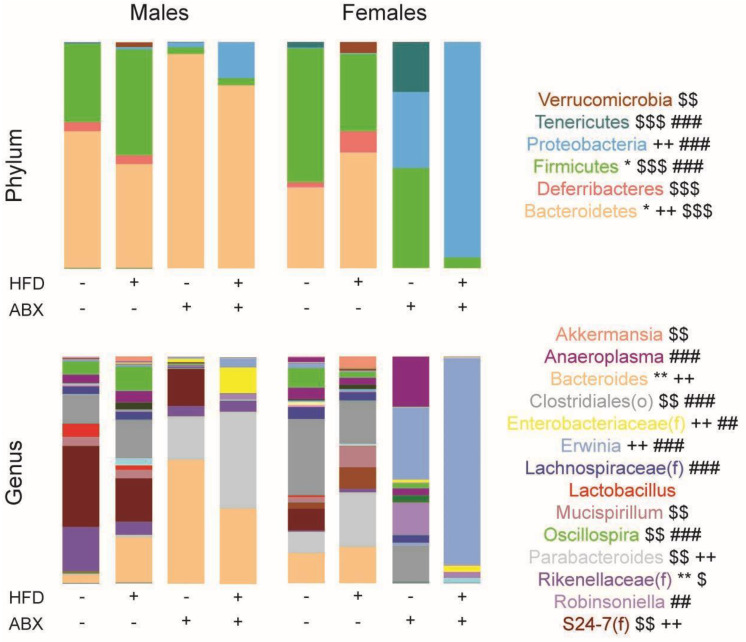
**Taxonomic composition varies by sex and is altered by high-fat diet (HFD) and antibiotics (Abx).** Taxonomic composition of the cecum by phylum and genus. Each color within the bar graph represents a phylum or genus and the colored area is proportional to the relative abundance. Levels of significance for phylum or genus are shown on the right. DESeq2 analysis was performed separately for males and females on phylum and genus level count data to identify taxa differentiating HFD vs. ND (no antibiotics) and HFD + Abx vs. ND + Abx. Males HFD vs. ND: * *p* < 0.05, ** *p* < 0.01. Females HFD vs. ND: $ *p* < 0.05, $$ *p* < 0.01, $$$ *p* < 0.001. Males HFD + Abx vs. ND + Abx: ++ *p* < 0.01. Females HFD + Abx vs. ND + Abx: ## *p* < 0.01, ### *p* < 0.001.

**Figure 7:**
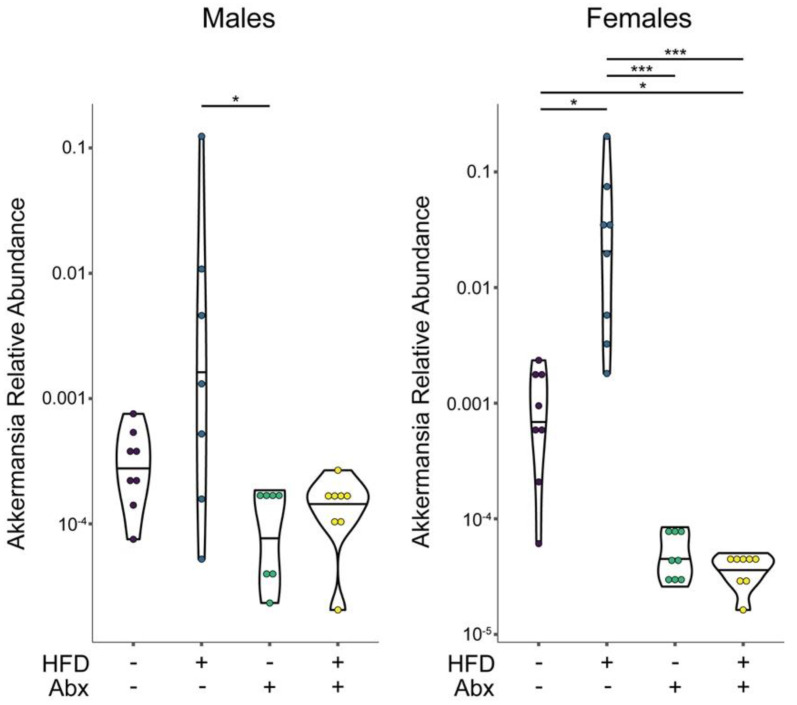
**High-fat diet induces *Akkermansia* to a greater extent in females than in males.** Violin plots showing relative abundance of *Akkermansia* in male and female mice, divided by dietary group (HFD = high-fat diet) and antibiotic group (Abx = antibiotic treated). Significance testing by Kruskal–Wallis with post-hoc Dunn’s test. * *p* < 0.05\*** *p* < 0.001.

There were significant changes in the composition of microbiome at the phylum and genus levels across the four treatment groups as well as between male and female mice (Figure 6). For instance, at the phylum level, HFD resulted in a lower relative abundance of Bacteroidetes and higher abundance of Firmicutes vs. ND in males, whereas it was the opposite in females. In addition, HFD induced a significant increase in Deferribacterium in females, which were undetected in ND or HFD with ABX. The addition of ABX to HFD increased the relative abundance of Proteobacteria in both sexes, particularly in combination with a HFD and more pronounced in females. Bacteroidetes also increased with ABX in males (Figure 6). In both males and females, HFD induced Verrucomicrobia, which were not detected in ND or in HFD with ABX; the increase in Verrucomicrobia in HFD vs. ND was significant in females only. At the genus level, HFD significantly increased *Bacteroides* and decreased unclassified Rikenellaceae in males but not females, whereas it increased the *Parabacteroides* and *Mucispirillum* in females but not in males. The addition of ABX increased the *Bacteroides* in both ND and HFD in males but not females. HFD + ABX increased unclassified Enterobacteriaceae and *Erwinia* vs. ND + ABX in both males and females, whereas the *Parabacteroides* were increased in HFD in females and in HFD + ABX in males. Only HFD induced *Akkermansia* in both males and females, but this reached statistically significance only in females (Figure 7).

**Figure 8:**
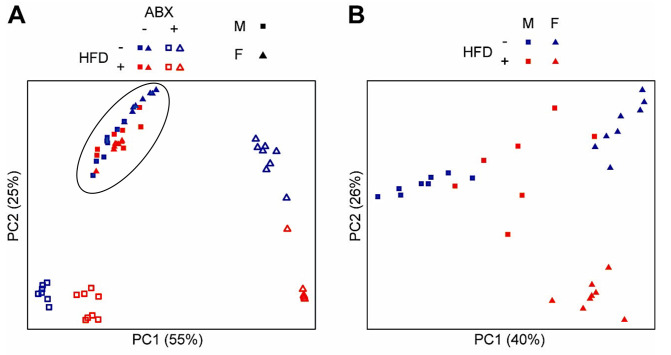
**Principal coordinates (PC) analysis plot based on weighted UniFrac distances showing effects of sex, antibiotics, and high-fat diet on microbial composition**. (**A**) Changes in microbial composition in high-fat diet (HFD) vs. normal diet (ND) without antibiotics (Abx; circle) and with Abx in males (M) and females (F). (**B**) Enlargement of data shown in (**A**) inside the circle illustrating changes in microbial composition in HFD vs. ND without Abx in males vs. females. Each symbol represents a mouse, with the color indicating the diet, the fill/not fill representing the presence or absence of Abx; and the shape indicating the sex. Note the separation in microbial composition in each group and in males and females. Males: *p* < 0.001; females: *p* < 0.001; *p* values for differences across groups are adjusted for diet, antibiotics, and sex.

Differences in microbial composition across experimental groups and sexes were visualized by Principal Coordinates Analysis based on weighted UniFrac distances (Figure 8). This analysis showed a complete separation of microbiota between females and males as well as between groups receiving different diets and in the presence or absence of ABX.

**Figure 9:**
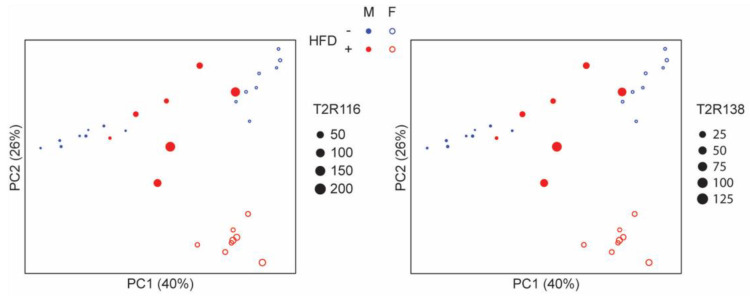
**Principal coordinates (PC) analysis plot showing association between microbiota composition and level of T2R expression in high-fat diet (HFD) that was more evident in the males**. Each symbol represents a mouse, with the color indicating the diet, the fill/not fill representing the sex. Dot size is proportional to the level of T2R expression.

Plotting microbial composition in each group and in males and females with the Tas2r138 or Tas2r116 mRNA expression confirms the separation by diet, sex, and ABX and shows that only HFD increases Tas2r138 and Tas2r116 mRNA expression indicating that Tas2r138 and Tas2r116 are induced in a microbiota-dependent manner (Figure 9).

**Figure 10:**
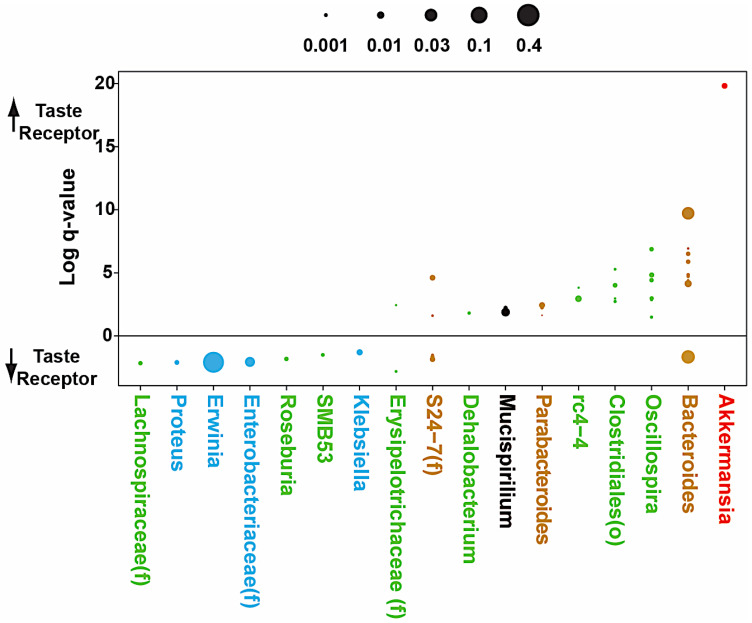
**Microbes associated with altered taste receptor expression.** Log q-values (*p*-values adjusted for multiple hypothesis testing) are shown for the association of operational taxonomic units (OTUs, roughly corresponding to species) with Tas2r expression in multivariate DESeq2 models adjusting for diet, antibiotics, and sex. Each dot represents 1 of 49 OTUs that were associated with both Tas2r116 and Tas2r138 with q < 0.05 and had normalized relative abundance greater than 0.0001. Dot size is proportional to the average normalized relative abundance of the OTU. Each dot represents an OTU. OTUs are arranged by genus and colored by phylum; (f) and (o) denote OTUs that could only be classified at the level of family or order.

Increased Tas2r138 and Tas2r116 mRNA levels in HFD were more pronounced in males. Differential abundance testing was then performed to identify individual microbes at the level of operational taxonomic units (OTUs, roughly corresponding to species). A combined analysis of males and females demonstrated 49 OTUs that were significantly associated with expression of both Tas2rs after adjusting for diet and ABX treatment (Figure 10).

**Figure 11:**
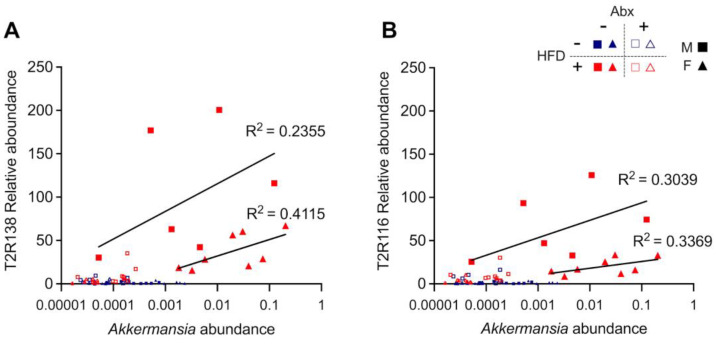
**Expansion of *Akkermansia muciniphila* on a high-fat diet is associated with upregulation of taste receptor expression**: *Akkermansia muciniphila* abundance and Tas2r138 (**A**) and Tas2r116 (**B**) (labeled T2R138 and T2R116 in the *y*-axis) expression for mice stratified by diet, antibiotic treatment, and sex. Separate regression lines are shown for males (M) and females (F) on a high-fat diet without antibiotics. Each symbol represents a mouse, with the color indicating the diet, the fill/not fill representing the presence or absence of Abx, and the shape indicating the sex.

Tas2r138 and Tas2r116 mRNA levels were most strongly associated with *Akkermansia muciniphila* followed by members of the *Bacteroides, Oscillospira, rc4-4* (within the Peptococcaceae family), *Parabacteroides*, and *Mucispirilium* genera and unclassified members of the Clostridiales order. Tas2r138 and Tas2r116 mRNA levels were negatively associated with multiple Proteobacteria including members of the *Proteus, Erwinia*, and *Klebsiella* genera as well as an unclassified Enterobacteriaceae. One highly abundant *Bacteroides* OTU was negatively associated with taste receptor expression. *Akkermansia muciniphila* abundance was found in linear regression analysis to be significantly correlated with Tas2r138 and Tas2r116 expression in both male and female mice fed a HFD (Figure 11).

## 4. Discussion

This study shows that a long-term HFD that induces obesity significantly reduced the density and variety of bacteria at the phylum and genus levels in the mouse large intestine concomitantly to a marked and significant increased level of expression of two distinct bitter taste receptors, the Tas2r138 and Tas2r116 mRNAs, with marked differences between males and females. By contrast, chronic treatment with ABX, which further reduced microbiome diversity and richness and markedly changed the microbiome composition, prevented the HFD-induced upregulation of Tas2r138 and Tas2r116. Furthermore, Tas2r138 and Tas2r116 mRNA levels were significantly correlated with different bacteria only in HFD fed mice without ABX treatment, suggesting a specific role of HFD-related, antibiotic independent, microbiota changes underlying the increased receptor expression.

The long-term HFD with or without ABX induced a significant increase in body weight and fat mass with a decrease in lean mass and an increase in glucose and lipid panel except for triglycerides consistent with the development of obesity and metabolic alterations, in both males and females. The incretins GLP-1 and GIP, and insulin were also increased by HFD, but only in males, perhaps in part reflecting the less pronounced increase in body weight and metabolic parameters observed in females vs. males. The different levels of GLP-1 and GIP might reflect different hormonal status of males and females [54]. Indeed, the higher levels of GLP-1 in females vs. males fed ND support hormonal driven differences. These different responses to the same HFD could also be ascribed to distinct microbiome composition that is diet- and sex-dependent [55,56,57], which is consistent with reported differences in the pathology of obesity depending in part on sex [58]. Interestingly, an increase in body weight and fat mass together with the concomitant decrease in lean mass occurred at 4 weeks, before the changes in the expression of Tas2r mRNAs became significant (8 weeks, this and our previous study [29]), suggesting that the upregulation of these taste receptors is more likely a consequence of the changes caused by the HFD, rather than a direct effect of the diet, though this needs to be proven. This is further supported by the suppression of Tas2r upregulation by ABX treatment that does not decrease body weight and further changes microbiome composition. The microbiome is important in maintaining energy homeostasis, and alteration of gut microbiome composition or dysbiosis is a major environmental factor in obesity [41,59], which precedes the chronic low grade inflammation and has been regarded as the initial stage of metabolic disorders [47,48,60]. The gut lumen is a major site of bacteria that are particularly abundant in the large intestine, and the symbiotic interaction between gut microbiota and the body is critical for nutrient absorption, energy harvesting, immune and metabolic functions, and the prevention of obesity and metabolic disorders. The association of microbiota and obesity is further supported by the observations that germ-free mice are protected from diet-induced obesity [61], genetically obese mice have impaired microbiota [62], and transfer of microbiota from obese mice induced obesity in lean, germ-free mice [63].

The analysis of the bacterial composition showed a significant decrease in Bacteriodetes with increase in Firmicutes in male mice induced by HFD, confirming previous findings [43]. However, this is reversed in male mice fed HFD plus ABX and is not observed in female mice that show the opposite with relative higher abundance of Bacteriodetes vs. Firmicutes in HFD. Furthermore, female mice show a completely different pattern of bacteria following HFD concomitantly with ABX with overwhelming abundance of Proteobacteria and *Erwinia*. Overall, there was a distinct microbiota separation in each of the four groups studied in males and females, which has not been previously appreciated. Both Tas2r138 and Tas2r116 were specifically induced in HFD fed mice in a microbiota-dependent manner, and were significantly associated, either positively or negatively, with bacteria belonging to 17 genera. Among these, there was *Akkermansia muciniphila*, a Gram-negative mucus-resident bacterium that seems to have a beneficial effect in metabolic, immune, and inflammatory diseases by attenuating intestinal mucosa damage and controlling intestinal homeostasis [64]. *Akkermansia muciniphila* was only observed following HFD, and it was significantly more pronounced in females vs. males. There was a positive and significant correlation between *Akkermansia muciniphila* and Tas2r138 and Tas2r116 in both males and females. While the molecular and cellular mechanisms underlying the effect of HFD on the Tas2rs and the microbiota remain to be defined, different hypotheses can be postulated. It is tempting to speculate that recognition of bacterial products by cells expressing Tas2r138 and Tas2r116 might be the trigger of signaling cascades that culminate in Tas2r gene regulation. Several lines of evidence support this hypothesis. Tas2rs138/TAS2R38 and Tas2r116/TAS2R16 are localized to EEC cells in rodents and human gut ([24,29], also Raybould and Sternini, unpublished observation), which upon stimulation release peptides/hormones such as CCK, GLP-1, and PYY, activating neuronal pathways that regulate GI functions and appetite [18,19]. In addition, Tas2r138 and its human counterpart TAS2R38 subtype have been shown to be activated by quorum-sensing molecules, such as acyl-homoserine lactones (AHL), produced by Gram-negative bacteria in the respiratory tract [37,38] and in an EEC cell line (Sternini and Rozengurt, unpublished observation). In the respiratory tract, AHL stimulation of TAS2R38 induces antimicrobial peptides and initiation of a defense response towards respiratory pathogens [37,38]. AHL is also produced by enteric pathogens in the gut with dysbiosis [65,66], which might activate Tas2r/TAS2R subpopulations in the gut, thus inducing a host response. Furthermore, bacteria-derived small molecules have been shown to alter host GI function by activating serotonin receptors and increasing fluid secretion, and microbiome alterations have been associated with biological effects of gut receptor signaling on nutrients and caloric intake [67,68]. Selective alterations in gut microbiota have also been shown to stimulate endogenous gut hormones production, with consequent beneficial effects on host physiology [69]. Moreover, chronic activation of a Tas2R subtype in vivo alters EEC hormone release and bile acid metabolism resulting in the amelioration of metabolic syndrome features [23]. Alternatively, it could be hypothesized that Tas2rs impact the microbial ecosystem in the gut by creating an environment favoring some microbes and disadvantaging others or that HFD induces host response resulting in increased taste receptor expression with concomitant effects on intestinal microbes.

## 5. Conclusions

The results of the current study showing upregulation of Tas2r138 and Tas2r116 in the large intestine of mice fed a long-term HFD and association with different bacteria strengthen the proposal that taste receptors are “sensors” of luminal bacteria [24,29]. Sensory receptors on EEC cells are critical for the integration of inputs from nutrients and non-nutrients, toxins, and bacteria within the body and are proposed as novel therapeutic targets for feeding disorders and metabolic diseases [13,25,33,70,71]. Though many questions remain to be addressed and further studies are required to establish causality in addition to association, the findings of this study provide support for a role of Tas2r138 and Tas2r116 in gut chemosensing and suggest that these bitter taste receptors could be explored as potential targets for treating diseases with microbiome disturbances.

## Data Availability

The authors confirm that all data underlying the findings are fully available. All relevant data are within the paper.

## Supplementary Materials

The following supporting information can be downloaded at: https://www.mdpi.com/article/10.3390/nu15194145/s1, Figure S1: Effect of high-fat diet (HFD) on Tas2r138 and Tas2r116 mRNA expression in the cecum mucosa of male mice, expressed as fold increase after 4 and 6 weeks of treatment.
