# Supplementary material for: Microbiota-Dependent Upregulation of Bitter Taste Receptor Subtypes in the Mouse Large Intestine in High-Fat Diet-Induced Obesity
Source: Nutrients. 2023 Sep 25;15(19):4145. doi: 10.3390/nu15194145 (PMC10574285; doi:10.3390/nu15194145)
Supplement: Supplementary file 1 [file nutrients-15-04145-s001.zip › Supplementary Figure S1.pdf]

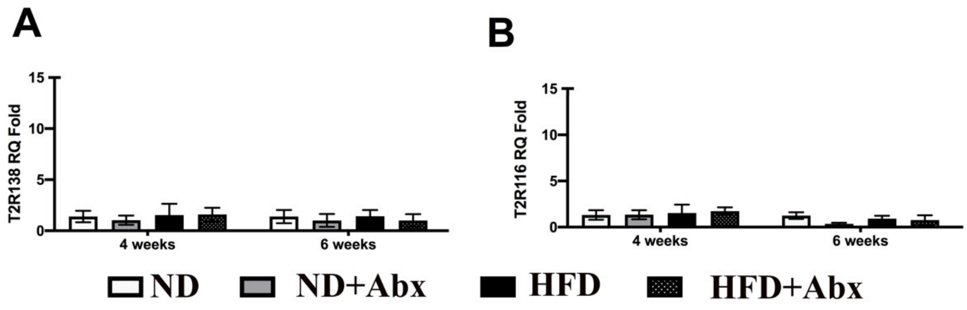

Supplementary Figure S1. Effect of high-fat diet (HFD) on Tas2r138 and Tas2r116 mRNA expression in the cecum mucosa of male mice, expressed as fold increase after 4 and 6 weeks of treatment. qRT-PCR analysis showed Tas2r138 mRNA (A) and Tas2r116 mRNA (B) expression in the cecum of male mice fed normal diet (ND), ND + antibiotics (Abx), HFD, or HFD +Abx (4 animals in each group for the 4 and 6 weeks). The levels of Tas2r138 and Tas2r116 mRNAs were comparable in the cecum of HFD with and without Abx compared to ND with or without Abx (A, B). Each column represents data from 4 mice. A total of 34 male mice were used for these experiments; 2 animals in the 6-weeks experiment had to be euthanized before the end of the experiment for health reasons; data for 32 animals are shown here.
